# ETV6 mutations define a new cancer predisposition syndrome

**DOI:** 10.18632/oncotarget.4842

**Published:** 2015-07-10

**Authors:** Gregory Kirkpatrick, Leila Noetzli, Jorge Di Paola, Christopher C. Porter

**Affiliations:** Department of Pediatrics, University of Colorado School of Medicine, Aurora, CO, USA

**Keywords:** Chromosome Section, leukemia, ETV6, predisposition, germline

Although acute lymphoblastic leukemia (ALL) is the most common childhood cancer, the etiology is usually unknown. However, genetic studies suggest that in some cases, inherited mutations in genes that regulate hematopoiesis contribute to leukemogenesis [1-3]. In a recent example, two independent studies of families with autosomal dominant thrombocytopenia and high incidence of lymphoblastic leukemia revealed multiple germline, single-nucleotide mutations in the gene encoding the ETS family transcriptional repressor ETV6 [2,3]. While *ETV6* is involved in the most common translocation found in childhood ALL (*ETV6-RUNX1*), and somatic *ETV6* mutations have also been previously described in B cell ALL [4], myelodysplastic syndrome [5] and T cell leukemias [6], these reports were the first to describe disease-causing germline *ETV6* mutations.

**Figure 1:**
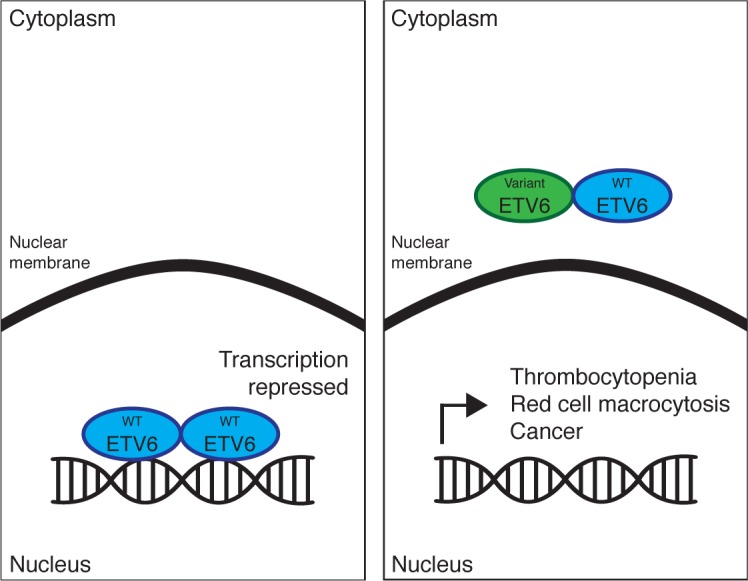
Mutation of ETV6 impairs nuclear localization and transcriptional repression

ETV6 contains three functional domains: pointed (PNT), involved in protein-protein interactions including homo-oligomerization; central, which promotes DNA binding and is essential for repressive function *in vitro;* and the ETS domain, which binds DNA. The two independent groups identified, four distinct mutations occurring in the ETS domain (R369Q, R399C, R418G, and 385_418del), and both groups identified the same mutation in the central domain (P214L). Functionally, each of these mutations appears to abrogate ETV6 nuclear localization, resulting in loss of transcriptional repression normally conferred by ETV6. Importantly, addition of WT ETV6 was unable to restore transcriptional repression lost by variant ETV6, suggesting that germline variant ETV6 acts in a dominant negative fashion (Figure 1). Impaired nuclear localization is common to all of the studied mutants, as well as ETV6-RUNX1 [7], implying a shared mechanism of failed tumor suppression. Interestingly, while in ETV6-RUNX1+ ALL the allele not involved in the translocation is usually deleted or mutated, the “wild-type” ETV6 allele remained intact in each of the four tumors studied by these groups, again supporting the hypothesis of dominant negative function of the inherited variant proteins.

Affected families studied by both groups displayed mild to moderate thrombocytopenia, some of them red cell macrocytosis, and predisposition not only for leukemia but also a number of other cancers. Malignancies developed in individuals inheriting ETV6 mutations included skin cancer, MDS, CMML, stage IV colon cancer, and, most commonly, pre-B cell ALL. Out of 17 patients carrying ETV6 variants studied by the University of Washington group, nine developed malignancies, including three leukemias. In 10 affected individuals studied by the University of Colorado group, three developed B cell leukemia. ETV6 mutations identified in both studies segregated completely with thrombocytopenia and predisposition toward cancer development, demonstrating a dominant transmission pattern. Interestingly, all patients with malignancies carried ETV6 mutations, but several individuals with mutations did not have cancer. Many somatic ETV6 mutations have been described previously in cancer, including somatic alterations affecting the same amino acids disrupted by recently described germline mutations. However, all five germline mutations identified were absent from public databases, and family members with inherited ETV6 variants did not exhibit mutations in other known leukemia predisposition genes, such as TP53, RUNX1 or ANKRD26. The consistent features of thrombocytopenia and cancer risk displayed by families in both studies highlights the functional relevance of germline ETV6 mutations, but the incomplete penetrance of malignancies indicates that other factors are required for oncogenesis.

The discovery of germline ETV6 variants is an exciting new finding with clinical relevance both for individuals inheriting these mutations as well as patients who may develop leukemia after acquiring mutations in ETV6 or related pathways. Little is currently known about what other oncogenic mediators may act in cooperation with mutated ETV6, but these factors likely play an important role in determining clinical outcome and may even represent targets for novel therapeutic approaches. Experiments to identify cooperating mutations using unbiased or targeted next generation sequencing identified alterations in a number of genes that are implicated in tumorigenesis, including RUNX1 and KRAS in a case of MDS, and a novel PAX5-SHB fusion in ALL. These data and those from large scale sequencing efforts such as TARGET, suggest that mutation in a variety of genes can cooperate with ETV6 dysfunction to promote leukemogenesis, particularly in those with critical roles in hematopoiesis.

In summary, these reports have established that ETV6 germline mutations impair its nuclear localization and transcriptional repression and result in thrombocytopenia and predisposition to cancer, but much remains to be explored in how ETV6 dysfunction initiates leukemogenesis, and what cooperating mutations are necessary for progression to clinical disease. The high incidence of B cell leukemias in these families, as well as the high rate of ETV6 translocation and/or deletion in sporadic precursor B cell ALL, suggests that ETV6 may play an unappreciated role in B cell development. The identification of dominant negative ETV6 variants will allow some of these questions to be addressed experimentally. Better understanding of aberrant ETV6 activity, as it relates to hematopoietic differentiation and leukemogenesis, may guide the development of new treatment strategies benefitting patients with both heritable and acquired forms of cancer.
